# Mitochondrial–neuroimmune interfaces in post-stroke spasticity: from acute brain injury to chronic motor phenotypes

**DOI:** 10.3389/fnins.2026.1927695

**Published:** 2026-08-05

**Authors:** Zihang Huang

**Affiliations:** School of Medical Technology, Tianjin University of Traditional Chinese Medicine, Tianjin, China

**Keywords:** mitochondria, mitochondrial quality control, neuroimmune signaling, neuroinflammation, post-stroke spasticity, reticulospinal tract, skeletal muscle remodeling, spinal inhibition

## Abstract

Post-stroke spasticity is a common and clinically consequential manifestation of the upper motor neuron syndrome, yet its mechanisms are incompletely explained by stretch reflex hyperexcitability alone. Established models emphasize corticospinal and corticoreticulospinal injury, altered brainstem descending drive, spinal reflex amplification, impaired inhibitory control, and secondary changes in skeletal muscle and connective tissue. In parallel, stroke induces profound mitochondrial stress and neuroimmune activation, including bioenergetic failure, mitochondrial reactive oxygen species production, mitochondrial quality-control disturbance, mitophagy dysregulation, mitochondrial danger signaling, glial activation, blood–brain barrier dysfunction, and peripheral immune responses. This Review examines how these mitochondrial–neuroimmune processes may interface with established neural and peripheral mechanisms to shape the onset, persistence, and heterogeneity of post-stroke spasticity. We distinguish strict reflex-mediated spasticity from broader spastic hypertonia, emphasizing that chronic clinical phenotypes often reflect mixed contributions from descending pathway imbalance, spinal disinhibition, spastic dystonia, passive muscle stiffness, pain, and contracture. We propose a brain–spinal cord–muscle framework in which mitochondrial and immune responses after stroke may modify motor-network plasticity, spinal inhibitory remodeling, skeletal muscle metabolism, autophagy-related tissue adaptation, and systemic inflammatory–metabolic vulnerability. Direct PSS-specific evidence remains limited. Accordingly, mitochondrial and neuroimmune pathways are framed here as candidate modifiers of phenotype trajectory rather than as established causes, validated biomarkers, or established therapeutic targets for PSS. The novelty of this Review lies in integrating established circuit and muscle mechanisms with broader stroke mitochondrial–immune biology to define testable interfaces and priorities for longitudinal phenotyping and mechanism-based trials.

## 1 Introduction

Post-stroke spasticity (PSS) is a common and clinically consequential component of the upper motor neuron syndrome. Although it is often recognized at the bedside as increased resistance to passive stretch, its impact extends beyond abnormal tone. In affected patients, spasticity may coexist with weakness, impaired selective motor control, abnormal synergies, pain, reduced range of motion, fatigue, soft-tissue shortening, and contracture. These combined impairments can interfere with walking, hand opening, dressing, hygiene, sleep, orthotic use, rehabilitation participation, and caregiver assistance. Population estimates vary according to stroke severity, timing of assessment, and diagnostic criteria; systematic reviews and longitudinal cohorts indicate that approximately one quarter of stroke survivors develop some degree of spasticity, whereas severe or disabling spasticity affects a smaller but clinically important subgroup (Lundström et al., 2008; Sommerfeld et al., 2004; Wissel et al., 2010; Zeng et al., 2021).

The classical definition of spasticity emphasizes a velocity-dependent increase in tonic stretch reflexes as part of the upper motor neuron syndrome (Lance, 1980). This definition remains valuable because it links spasticity to stretch reflex hyperexcitability, spinal sensorimotor processing, and descending motor control. However, post-stroke hypertonia in clinical practice is rarely explained by reflex exaggeration alone. Resistance to passive movement can also reflect spastic dystonia, involuntary resting muscle activity, abnormal co-contraction, pain-related guarding, altered muscle architecture, increased extracellular matrix stiffness, tendon shortening, and fixed contracture (Dietz and Sinkjær, 2007; Trompetto et al., 2014, 2019). For this reason, PSS is best approached as a heterogeneous and temporally evolving motor phenotype rather than a single reflex abnormality.

Established mechanistic models have focused primarily on the neural consequences of supraspinal injury. Damage to corticospinal and corticoreticulospinal projections reduces voluntary motor control and alters the balance between descending inhibitory and facilitatory influences on spinal motor circuits (Ko et al., 2021; Lee-Hotta, 2026; Li and Francisco, 2015; Li, 2017). Increased reliance on brainstem descending pathways, particularly reticulospinal output, may support some forms of gross motor recovery but can also reinforce abnormal synergies, impaired fractionation, and excessive muscle activation (Choudhury et al., 2019; Ko et al., 2021; Lee-Hotta, 2026; Li and Francisco, 2015; Li, 2017). At the spinal level, altered sensory afferent input, reduced inhibitory processing, impaired presynaptic or reciprocal inhibition, and increased motoneuron excitability can amplify stretch reflex responses (Burke et al., 2013; Katz and Rymer, 1989; Lamy et al., 2005, 2009; Sheean and McGuire, 2009). This neural framework remains the most secure foundation for understanding PSS.

Yet neural circuit models alone do not fully explain the delayed onset, variable severity, phenotype diversity, or progressive tissue-level consequences of PSS. Some patients develop mild stretch hyperreflexia without major functional impact, whereas others progress toward painful, disabling, contracture-prone hypertonia. Similar lesion burdens may produce different spastic patterns depending on corticoreticular involvement, spinal excitability, immobility, systemic metabolic state, rehabilitation exposure, and skeletal muscle adaptation. These observations suggest that PSS is shaped not only by the initial lesion and descending pathway disruption, but also by the biological environment in which motor circuits and peripheral tissues remodel after stroke.

Mitochondrial stress and neuroimmune activation are central components of this post-injury environment. Cerebral ischemia and hemorrhagic injury disrupt oxidative phosphorylation, deplete ATP, increase intracellular calcium load, generate mitochondrial reactive oxygen species, impair mitochondrial quality control, and activate cell-death pathways (Gao et al., 2024; Liu et al., 2018; Qin et al., 2022; Yang et al., 2022). Damaged mitochondria can also function as inflammatory organelles by releasing or exposing mitochondrial danger signals, including mitochondrial DNA, oxidized mitochondrial DNA, cardiolipin, cytochrome c, and other damage-associated molecular patterns. These signals can engage innate immune pathways such as NLRP3 inflammasome activation and cGAS–STING signaling, thereby linking mitochondrial injury to sterile inflammation (Bellut et al., 2023; Billingham et al., 2022; Kong et al., 2022; McArthur et al., 2018; Rongvaux et al., 2014; Shimada et al., 2012; West et al., 2015; White et al., 2014; Zhong et al., 2018; Zhou et al., 2011). In parallel, microglia, astrocytes, endothelial cells, pericytes, infiltrating leukocytes, and systemic immune responses shape blood–brain barrier integrity, synaptic remodeling, axonal repair, white matter recovery, and chronic neuroinflammatory states (Iadecola and Anrather, 2011; Iadecola et al., 2020; O’Reilly and Tom, 2020; Qiao et al., 2023; Shi et al., 2019; Zhao et al., 2025).

These mitochondrial–neuroimmune mechanisms are well established in stroke biology, but their relationship to PSS remains underexplored. Direct evidence that mitochondrial dysfunction, mitophagy disturbance, mitochondrial DNA release, NLRP3 activation, STING signaling, or glial metabolic reprogramming causes clinical spasticity is currently limited. A more defensible and biologically useful formulation is that mitochondrial–neuroimmune processes may act as upstream modifiers of phenotype trajectory. By influencing neuronal survival, glial state, vascular stability, synaptic remodeling, spinal inhibitory tone, immune-cell metabolism, skeletal muscle bioenergetics, and tissue stiffness, these processes may help determine whether post-stroke plasticity evolves toward functional recovery, maladaptive reflex amplification, or chronic mixed neural–non-neural hypertonia.

The skeletal muscle compartment is particularly important in this framework. Hemiparetic muscle after stroke can undergo atrophy, altered fiber-type composition, reduced oxidative capacity, intramuscular fat accumulation, autophagy-related changes, increased passive stiffness, and extracellular matrix remodeling (Azzollini et al., 2021; Beckwée et al., 2022; Hafer-Macko et al., 2008; Qi et al., 2023; Severinsen et al., 2016; Stecco et al., 2014). These changes do not constitute spasticity in the strict reflex-mediated sense, but they can amplify the clinical expression of spastic hypertonia by increasing passive resistance, reducing tissue compliance, promoting contracture, and limiting rehabilitation responsiveness. Thus, PSS should be considered across a brain–spinal cord–muscle axis in which descending motor pathway imbalance, spinal reflex remodeling, and peripheral tissue adaptation interact over time.

The novelty of this Review lies in integrating established neural and peripheral mechanisms of PSS with broader mitochondrial and neuroimmune biology after stroke. We organize the evidence across a brain–spinal cord–muscle interface and explicitly distinguish direct PSS evidence from broader stroke and comparator-model evidence. This framework is intended to identify testable modifiers of phenotype trajectory, not to propose a new primary cause of spasticity.

### 1.1 Literature search and scope of the review

This article is a narrative Review designed to integrate evidence from post-stroke spasticity, stroke mitochondrial biology, neuroimmune regulation, spinal reflex physiology, and skeletal muscle remodeling. We searched PubMed/MEDLINE, Web of Science, Scopus, and Google Scholar for English-language articles published through July 1, 2026, using combinations of the following terms: “post-stroke spasticity,” “spastic hypertonia,” “upper motor neuron syndrome,” “reticulospinal tract,” “corticoreticulospinal pathway,” “spinal inhibition,” “KCC2,” “H-reflex,” “stroke mitochondria,” “mitochondrial quality control,” “mitophagy,” “mitochondrial DNA,” “NLRP3 inflammasome,” “cGAS-STING,” “neuroinflammation,” “microglia,” “astrocytes,” “blood–brain barrier,” “skeletal muscle after stroke,” “muscle stiffness,” and “shear-wave elastography.” Priority was given to peer-reviewed original studies, systematic reviews, meta-analyses, clinical statements, and mechanistic reviews. We included classic papers that established key concepts in spasticity and upper motor neuron physiology, as well as recent studies addressing mitochondrial stress, neuroimmune activation, and post-stroke motor or muscle phenotypes. Study selection was purposive rather than exhaustive. We prioritized studies with direct relevance to clinically or experimentally defined PSS, clear mechanistic relevance to the proposed interfaces, or landmark methodological value. Evidence was grouped as direct human PSS evidence, post-stroke preclinical evidence with spasticity-related outcomes, broader stroke evidence without a PSS-specific endpoint, or comparator-model evidence. Comparator studies were used only to support biological plausibility. The search was limited to English-language sources; peer-reviewed full-text articles were prioritized, and conference abstracts without a full article were not used as key evidence. Landmark book chapters and clinical statements were retained when they established key concepts. Most mechanistic evidence came from ischemic stroke, so extrapolation to hemorrhagic stroke was cautious. No formal risk-of-bias assessment or pooled analysis was performed.

## 2 Clinical phenotype, terminology, and temporal evolution of post-stroke spasticity

### 2.1 Why terminology matters

Post-stroke spasticity (PSS) is often used as a convenient clinical label for increased resistance to passive movement, but the term can obscure substantial biological heterogeneity. A limb that feels “tight” after stroke may reflect stretch reflex hyperexcitability, involuntary tonic activity, abnormal descending drive, impaired reciprocal inhibition, pain-related guarding, passive muscle stiffness, tendon shortening, or fixed contracture. These processes frequently coexist, but they are not mechanistically equivalent. A review that aims to connect PSS with mitochondrial–neuroimmune mechanisms must therefore begin by defining the phenotype with sufficient precision.

The preferred umbrella term in this Review is PSS when referring to the clinical syndrome and literature, and spastic hypertonia when emphasizing the mixed neural and non-neural sources of resistance. The term muscle spasm should be avoided as the main descriptor because it implies episodic involuntary contractions and does not accurately capture the chronic, stretch-sensitive, and motor-control-related phenotype of PSS. When spasm is used, it should refer specifically to intermittent involuntary contractions rather than the whole syndrome.

### 2.2 Post-stroke spasticity within the upper motor neuron syndrome

Post-stroke spasticity (PSS) should be interpreted within the broader upper motor neuron syndrome rather than as an isolated sign. Negative features include weakness, loss of dexterity, reduced selective motor control, slowed movement, and fatigability; positive features include spasticity, exaggerated reflexes, clonus, spasms, spastic dystonia, and abnormal synergies. This distinction matters because the clinical burden of PSS often depends less on tone alone than on its interaction with weakness, poor voluntary control, pain, and reduced range of motion.

A patient with severe wrist and finger flexor hypertonia may have several superimposed contributors: reduced corticospinal drive to extensors, abnormal flexor synergy, stretch-evoked reflex activity, resting flexor activation, soft-tissue shortening, and pain during hand opening. A patient with equinovarus posture may similarly have plantarflexor/invertor overactivity, impaired dorsiflexor activation, altered sensory feedback, Achilles tendon shortening, and stiffness of the muscle–tendon unit. These phenotypes cannot be understood by a single Modified Ashworth Scale score or by a single pathway.

### 2.3 Strict spasticity versus spastic hypertonia

A central distinction in this manuscript is between strict spasticity and spastic hypertonia. Strict spasticity refers to exaggerated stretch reflex activity in a relaxed muscle, typically expressed as velocity-dependent resistance to passive stretch. By contrast, spastic hypertonia is a broader clinical construct that includes strict spasticity but may also include spastic dystonia, abnormal co-contraction, associated reactions, intrinsic muscle stiffness, connective tissue remodeling, tendon shortening, and fixed contracture (Dietz and Sinkjær, 2007; Trompetto et al., 2014).

This distinction is critical when discussing skeletal muscle mitochondria. Muscle mitochondrial dysfunction, reduced oxidative capacity, autophagy-related remodeling, fibrosis, and passive stiffness should not be described as spasticity in the strict neurophysiological sense. They are better understood as contributors to the non-neural component of spastic hypertonia.

### 2.4 Spastic dystonia, spasms, and contracture

Spastic dystonia deserves explicit attention because it is frequently hidden under the umbrella of spasticity. It refers to involuntary tonic muscle contraction in the context of an upper motor neuron lesion and can occur even in the absence of a stretch trigger. In stroke subjects with increased wrist flexor tone, spontaneous and stretch-sensitive EMG activity has been reported in many hypertonic muscles, supporting the idea that resting overactivity may contribute substantially to post-stroke hypertonia (Trompetto et al., 2019).

Spasms are episodic involuntary contractions, often triggered by movement, posture, discomfort, infection, or other stimuli. Contracture refers to fixed limitation of passive range of motion due to changes in muscle, tendon, capsule, fascia, or periarticular tissues. Stretch-evoked spasticity, resting spastic dystonia, episodic spasms, and fixed contracture may all increase resistance or abnormal posture after stroke, but they differ in trigger, physiology, measurement, and treatment responsiveness.

### 2.5 Epidemiology, burden, and temporal evolution

Reported prevalence of PSS varies widely because studies differ in stroke severity, inclusion of paresis, timing after stroke, assessment tool, threshold for spasticity, and whether disabling spasticity is separated from any increase in tone. A systematic review and meta-analysis reported a pooled PSS prevalence of 25.3%, a prevalence of 26.7% after first-ever stroke, and an incidence of 39.5% among patients with first-ever stroke and paresis; severe or disabling spasticity affected 9.4% of stroke patients with paresis (Zeng et al., 2021). Earlier cohort studies similarly show that PSS is common but unevenly disabling (Lundström et al., 2008; Sommerfeld et al., 2004; Wissel et al., 2010).

The timing of PSS is central to this Review. Spasticity is not always present immediately after stroke; it often evolves during the subacute period as descending pathway imbalance, spinal reflex remodeling, reduced voluntary control, immobility, and muscle changes accumulate. Wissel and colleagues found that increased muscle tone could appear within the first weeks and that spasticity was associated with pain and worse activity or quality-of-life measures in their cohort (Wissel et al., 2010). This delayed consolidation creates a conceptual window in which early biological injury, maladaptive circuit plasticity, reduced activity, and peripheral tissue remodeling may interact before chronic hypertonia becomes fixed.

### 2.6 Clinical phenotypes relevant to a mechanism-oriented review

A mechanism-oriented Review should not treat all PSS as one phenotype. Early reflex-dominant spasticity is most directly linked to descending disinhibition and spinal reflex amplification. Upper-limb flexor-dominant spasticity may be especially relevant to corticoreticulospinal imbalance and reticulospinal contribution. Lower-limb extensor or equinovarus-dominant spasticity can include neural overactivity, altered postural control, tendon shortening, and passive stiffness. Spastic dystonia-dominant hypertonia may be missed if assessment focuses only on passive stretch. Painful and contracture-dominant phenotypes require attention to nociceptive and tissue-level mechanisms. Fatigue or metabolic vulnerability phenotypes may be particularly relevant to the mitochondrial–muscle axis.

These categories are not mutually exclusive. A patient may progress from early reflex hyperexcitability to mixed reflex and muscle stiffness, and later to contracture-dominant hypertonia. This evolution is one reason that clinical phenotype must be linked with time after stroke and with neural, muscular, and systemic mechanisms. The clinical phenotypes used to structure this Review are summarized in Table 1.

**TABLE 1:** Clinical phenotypes within the post-stroke spasticity and hypertonia spectrum.

Clinical phenotype	Typical presentation	Dominant mechanisms to consider	Mechanism-informed assessments
Reflex-dominant spasticity	Velocity-dependent resistance, stretch-evoked catch, brisk reflexes or clonus	Loss of descending inhibition, increased spinal reflex gain, altered afferent processing	Modified Tardieu Scale, stretch-evoked EMG, H-reflex
Synergy-dominant spastic motor phenotype	Upper-limb flexor synergy, lower-limb extensor or equinovarus pattern, effort-related co-activation	Corticospinal injury, corticoreticulospinal imbalance, increased brainstem-mediated descending drive	CST/CRT imaging, EMG during voluntary tasks, StartReact where feasible
Spastic dystonia-dominant hypertonia	Involuntary tonic activity at rest, persistent abnormal posture despite relaxation	Abnormal descending drive, impaired inhibitory control, resting motoneuron activation	Resting EMG, clinical observation, response to focal chemodenervation
Muscle stiffness-dominant hypertonia	High passive resistance, reduced range of motion, stiffness disproportionate to stretch-evoked activity	Muscle shortening, extracellular matrix remodeling, fibrosis, tendon stiffness, disuse	Ultrasound, shear-wave elastography, passive torque, range-of-motion measures
Painful or care-limiting hypertonia	Hypertonia associated with pain, hygiene difficulty, sleep disturbance, or caregiver burden	Mixed neural overactivity, soft-tissue pain, abnormal posture, contracture risk	Pain scales, goal attainment, functional and caregiver-related outcomes
Severe mixed phenotype	Disabling mixed hypertonia with weakness, poor voluntary control, reflex overactivity, stiffness, and contracture risk	Combined descending imbalance, spinal reflex amplification, spastic dystonia, muscle remodeling, systemic modifiers	Multimodal assessment combining clinical scales, EMG, imaging, elastography, pain, and function

CST, corticospinal tract; CRT, corticoreticular tract; EMG, electromyography.

### 2.7 Implications for the mitochondrial–neuroimmune framework

Because PSS is heterogeneous and evolves over time, mitochondrial–neuroimmune processes are unlikely to relate equally to all phenotype components. They may modify motor-network plasticity, spinal inhibitory tone, muscle bioenergetics and stiffness, pain, and rehabilitation responsiveness. This framework therefore requires phenotype-specific and time-sensitive testing rather than assuming that any single mitochondrial or immune pathway directly increases stretch-reflex gain.

## 3 Established neural mechanisms: descending pathway imbalance and spinal reflex hyperexcitability

### 3.1 Neural mechanisms remain the foundation

Post-stroke spasticity (PSS) is most securely understood as a consequence of disturbed descending motor control and altered spinal reflex processing. Although the clinical syndrome is recognized by increased resistance to passive stretch, the underlying biology is distributed across the motor cortex, corticospinal and corticoreticular projections, brainstem descending systems, spinal interneuronal networks, motoneurons, sensory afferents, and peripheral muscles. This distributed organization is important because spasticity is not simply the passive expression of a lesion; it evolves over days to weeks after stroke and reflects plastic changes in surviving neural circuits (Lee-Hotta, 2026).

The established neural model has two complementary components. The first is supraspinal imbalance: stroke disrupts corticospinal and corticoreticulospinal control, reducing voluntary drive and altering the balance between descending inhibitory and facilitatory influences on spinal reflexes. The second is spinal amplification: altered sensory afferent gain, impaired inhibitory processing, and increased motoneuron responsiveness increase the probability that stretch, movement, or voluntary effort will evoke excessive muscle activation (Burke et al., 2013; Choudhury et al., 2019; Katz and Rymer, 1989; Ko et al., 2021; Lamy et al., 2005, 2009; Li and Francisco, 2015; Li, 2017; Sheean and McGuire, 2009).

### 3.2 Corticospinal injury and impaired voluntary motor control

Corticospinal tract injury is central to post-stroke motor impairment, particularly weakness, reduced fractionated movement, impaired dexterity, and loss of selective voluntary control. However, corticospinal damage alone does not fully explain the development or severity of spasticity. Patients with severe corticospinal injury may show profound weakness with variable degrees of hypertonia, whereas others develop prominent spastic synergies, exaggerated reflexes, and involuntary co-activation. This dissociation indicates that PSS is not merely the motor expression of corticospinal tract loss, but also reflects how remaining descending systems and spinal circuits reorganize after injury.

### 3.3 Corticoreticulospinal imbalance and enhanced reticulospinal influence

Among descending systems, the corticoreticulospinal pathway has become particularly important in contemporary models of PSS. Cortical injury and corticoreticular disruption can disinhibit brainstem reticular formation circuits, increasing the influence of reticulospinal projections on spinal motor networks (Ko et al., 2021; Lee-Hotta, 2026; Li and Francisco, 2015; Li, 2017). Reticulospinal projections are strongly involved in posture, gross limb movements, axial and proximal control, and stereotyped motor patterns. After corticospinal damage, increased reliance on reticulospinal output may support some forms of gross motor recovery but can also reinforce abnormal synergies and spastic overactivity.

Human evidence for corticoreticulospinal involvement is growing but remains imperfect. Diffusion tensor imaging studies have examined the corticoreticular pathway in relation to PSS, (Ko et al., 2021) and StartReact paradigms provide indirect evidence for altered reticulospinal output (Choudhury et al., 2019). Reticulospinal involvement should therefore be framed as a major contributor, not a universal explanation. Upper-limb flexor synergies, impaired hand individuation, and exaggerated startle-associated activation may be particularly compatible with enhanced reticulospinal influence, whereas lower-limb extensor patterns may also involve vestibulospinal, spinal, and peripheral mechanisms.

### 3.4 Spinal reflex amplification and impaired inhibitory control

At the spinal level, PSS reflects increased responsiveness of the stretch reflex pathway and related sensorimotor circuits. Multiple mechanisms can contribute: increased Ia afferent input, reduced presynaptic inhibition, impaired reciprocal inhibition, altered recurrent inhibition, changes in post-activation depression, enhanced polysynaptic reflex transmission, and increased motoneuron excitability (Lamy et al., 2005; Lamy et al., 2009). These processes are not independent of supraspinal injury; descending pathways normally regulate the excitability of spinal interneurons and motoneurons, and after stroke altered descending drive can shift spinal circuits toward a more excitable state.

Spasticity and motor recovery both involve plasticity, but they are not the same process. Recovery requires reorganization of surviving cortical, subcortical, brainstem, spinal, and peripheral systems to restore movement or develop compensation. Spasticity may emerge when plasticity increases involuntary coupling, reflex gain, and non-selective descending drive rather than restoring fractionated voluntary control (Li, 2017). The key question for later sections is whether mitochondrial stress and neuroimmune activation modify this direction of plasticity.

### 3.5 What the neural model explains and what it leaves unresolved

**FIGURE 1:**
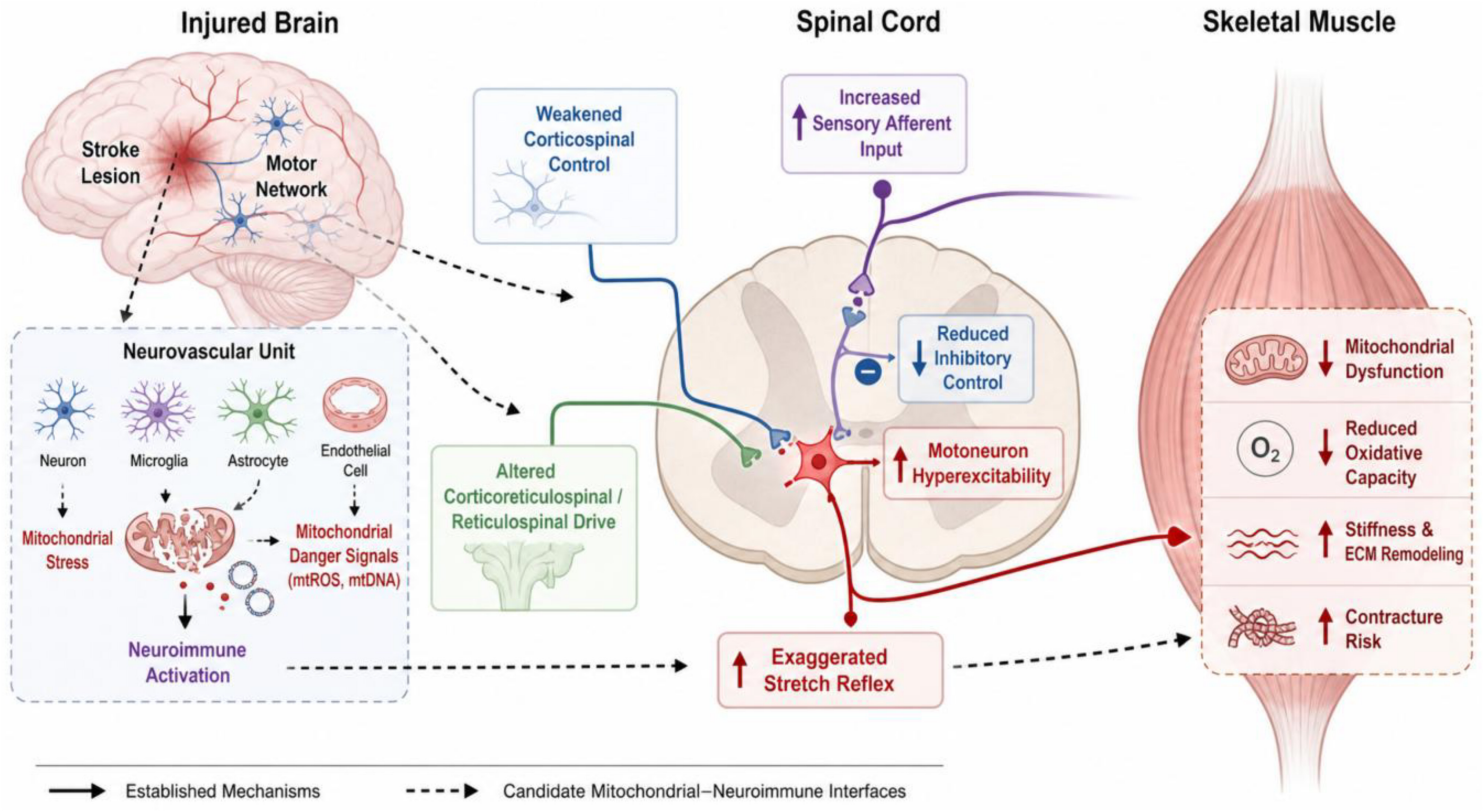
Proposed brain–spinal cord–muscle interface model in post-stroke spastic motor phenotypes. Stroke induces mitochondrial stress and neuroimmune activation within the injured neurovascular unit. These processes may modify the recovery environment in which descending motor pathways and spinal reflex circuits remodel. Established mechanisms of post-stroke spasticity include weakened corticospinal control, altered corticoreticulospinal and reticulospinal drive, spinal disinhibition, increased sensory afferent input, and motoneuron hyperexcitability. Over time, skeletal muscle remodeling, including mitochondrial dysfunction, reduced oxidative capacity, stiffness, extracellular matrix remodeling, and contracture risk, may amplify the non-neural component of spastic hypertonia. Solid colored arrows indicate better-established mechanisms; dashed arrows indicate plausible but incompletely tested mitochondrial–neuroimmune interfaces. Muscle mitochondrial and structural changes are depicted as contributors to non-neural hypertonia and contracture risk, not as direct causes of reflex-mediated spasticity.

The established neural model explains the association of PSS with upper motor neuron injury, its stretch-evoked nature, its relationship to altered descending control, its coupling with abnormal synergies, and its dependence on spinal reflex excitability. However, it does not fully explain why similar lesions lead to different spastic phenotypes, why some patients develop disabling contracture-dominant hypertonia whereas others do not, or how acute stroke biology influences chronic motor outcomes. Mitochondrial injury, glial and immune responses, blood–brain barrier disruption, systemic inflammation, and skeletal muscle metabolic remodeling should therefore be considered candidate modifiers of the environment in which neural circuits and muscles remodel after stroke. These relationships are integrated conceptually in Figure 1.

## 4 Mitochondrial stress and neuroimmune activation after stroke

### 4.1 Mitochondrial injury as an organizer of post-stroke tissue responses

Stroke imposes an immediate energetic crisis on the neurovascular unit. Interruption of oxygen and glucose supply disrupts oxidative phosphorylation, depletes ATP, collapses ionic gradients, enhances glutamate excitotoxicity, increases intracellular calcium load, and promotes mitochondrial reactive oxygen species generation (Gao et al., 2024; Qin et al., 2022; Yang et al., 2022). These events are traditionally discussed as upstream mechanisms of neuronal death, but they also influence glial activation, endothelial integrity, blood–brain barrier disruption, innate immune signaling, and the repair environment in which motor networks reorganize after injury.

This broader view is important for PSS. The established circuit model explains how corticospinal and corticoreticulospinal disruption can alter descending control and spinal reflex gain, but it does not fully address the cellular milieu in which these circuit changes unfold. Mitochondrial stress may shape this milieu by regulating neuronal survival, synaptic maintenance, glial phenotype, axonal repair, vascular stability, and immune-cell behavior. The degree and duration of mitochondrial stress are likely to matter: moderate responses may activate adaptive programs, whereas sustained or excessive stress can damage tissue integrity and amplify inflammation (Gao et al., 2024). These mechanisms are well established in stroke injury biology, but their relevance to clinically defined PSS remains indirect.

### 4.2 Mitochondrial quality control: dynamics, mitophagy, and biogenesis

Mitochondrial quality control provides a structured way to connect acute ischemic injury with longer-term cellular adaptation. Cells maintain mitochondrial homeostasis through coordinated fission and fusion, selective mitophagy, mitochondrial biogenesis, proteostasis, and metabolic remodeling. After stroke, these processes can determine whether injured neurons, astrocytes, microglia, endothelial cells, oligodendrocytes, and infiltrating immune cells preserve functional mitochondria or accumulate dysfunctional organelles that perpetuate oxidative stress and inflammatory signaling (Yang et al., 2022).

Mitochondrial dynamics are not simply morphological events. Excessive fission, often associated with Drp1 activation, can fragment mitochondria and promote apoptosis or inflammatory signaling; fusion proteins such as Mfn1, Mfn2, and OPA1 help maintain mitochondrial network integrity and respiratory function. Mitophagy is equally important but difficult to interpret simplistically: insufficient mitophagy can allow damaged mitochondria to accumulate, whereas excessive or poorly timed mitophagy may compromise bioenergetics if mitochondrial removal exceeds replacement. Accordingly, mitochondrial quality-control pathways should be regarded as hypothesis-generating modifiers of the post-stroke recovery environment rather than as PSS-specific mechanisms.

### 4.3 Neuroimmune activation after stroke

The immune response to stroke begins within the injured brain but rapidly becomes a distributed process involving resident glial cells, vascular cells, infiltrating leukocytes, lymphoid organs, and systemic immune regulation. Foundational and updated stroke immunology reviews emphasize that innate and adaptive immune responses contribute to acute tissue injury, post-stroke infection risk, repair, and therapeutic opportunities (Iadecola and Anrather, 2011; Iadecola et al., 2020). Microglia respond rapidly by changing morphology, metabolism, phagocytic activity, cytokine production, and interactions with astrocytes, neurons, endothelial cells, and infiltrating immune cells. Astrocytes contribute to cytokine signaling, glutamate handling, antioxidant support, scar formation, and blood–brain barrier regulation.

The temporal profile of inflammation is critical. Acute inflammation can worsen injury, but later immune and glial responses also participate in debris clearance, angiogenesis, synaptic remodeling, remyelination, and repair. Global brain inflammation after stroke has been proposed to persist beyond the focal lesion and to shape evolving pathology and long-term neurological outcome (Shi et al., 2019). Recent evidence further suggests that chronic microglial metabolic states, including cholesterol metabolic reprogramming, can sustain post-stroke neuroinflammation and hinder neurorestoration (Zhao et al., 2025). These observations support a role for immune state in long-term stroke recovery, but they do not establish a PSS-specific immune pathway. Their relevance to PSS therefore lies in the possibility that persistent immune-metabolic states modify motor-network plasticity, spinal inhibitory remodeling, or phenotype heterogeneity.

### 4.4 Damaged mitochondria as inflammatory organelles

Mitochondria can act as inflammatory organelles when bioenergetic failure and structural damage expose or release mitochondrial molecules that are interpreted as danger signals. Mitochondrial ROS, oxidized mitochondrial DNA, cardiolipin, cytochrome c, N-formyl peptides, and other mitochondrial danger-associated molecular patterns can activate innate immune pathways. This is the molecular bridge that most directly links mitochondrial injury to neuroimmune activation in stroke.

The NLRP3 inflammasome is one of the best-studied mitochondrial–immune interfaces. Foundational work showed that blockade of autophagy and mitophagy leads to accumulation of damaged ROS-generating mitochondria and activation of NLRP3; subsequent work linked oxidized mitochondrial DNA, mitochondrial DNA synthesis, and electron transport chain function to inflammasome activation (Billingham et al., 2022; McArthur et al., 2018; Shimada et al., 2012; Zhong et al., 2018; Zhou et al., 2011). Mitochondrial DNA also links mitochondrial injury to cGAS–STING signaling and type I interferon responses (Rongvaux et al., 2014; West et al., 2015; White et al., 2014). In ischemic stroke models, mtDNA–STING signaling has been implicated in microglial polarization, and delayed NLRP3 inhibition has been reported to ameliorate subacute stroke progression in mice (Bellut et al., 2023; Kong et al., 2022).

These pathways should be positioned upstream of PSS rather than as direct PSS mechanisms. NLRP3, STING, mtDNA, and related mitochondrial danger pathways are best described as candidate modifiers of the post-stroke inflammatory environment in which motor circuits and muscles remodel. Table 2 summarizes the main mitochondrial–neuroimmune interfaces according to evidence directness and plausible relevance to PSS.

**TABLE 2:** Mitochondrial–neuroimmune interfaces potentially relevant to post-stroke spasticity.

Mechanistic interface	Main evidence base	Plausible relevance to PSS	Evidence strength / main PSS-specific gap
Mitochondrial bioenergetic failure and oxidative stress	Strong evidence in acute stroke biology	May influence neuronal survival, glial activation, vascular injury, and the recovery environment in which motor circuits remodel	IndirectMain PSS-specific gap: No longitudinal human PSS studies controlling for lesion severity and paresis.
Mitochondrial quality control and mitophagy	Strong preclinical and review evidence in stroke models	May regulate persistence or resolution of mitochondrial stress, inflammatory signaling, and tissue repair	IndirectMain PSS-specific gap: No human PSS studies and few stroke models with spasticity-related outcomes.
Mitochondrial danger signaling	Strong mechanistic evidence for mtDNA, mtROS, NLRP3, and cGAS-STING pathways; emerging stroke evidence	May link mitochondrial injury to sterile inflammation, microglial activation, and subacute inflammatory remodeling	Indirect; PSS-specific evidence limitedMain PSS-specific gap: No PSS-specific predictive evidence independent of overall stroke severity.
Neuroimmune and glial metabolic remodeling	Strong stroke immunology evidence; emerging evidence for chronic microglial metabolic states	May shape synaptic remodeling, white matter repair, motor-network plasticity, and recovery trajectory	Plausible but unprovenMain PSS-specific gap: No cell- or compartment-specific link to PSS-related motor or spinal physiology.
Brainstem-spinal inhibitory remodeling	Direct but limited stroke animal evidence; stronger comparator evidence from spinal cord injury	May connect supraspinal injury to spinal disinhibition, altered chloride homeostasis, increased afferent input, and reflex amplification	Moderate preclinical; limited human evidenceMain PSS-specific gap: No confirmation of KCC2-related disinhibition in human PSS.
Skeletal muscle mitochondrial and mechanical remodeling	Human and animal evidence for post-stroke muscle metabolic and structural changes	May contribute to fatigue, stiffness, reduced compliance, contracture risk, and non-neural spastic hypertonia	Moderate for muscle remodeling; limited for strict spasticityMain PSS-specific gap: Few paired studies separate passive stiffness from stretch-evoked neural activity.

Evidence for mitochondrial and neuroimmune mechanisms is strongest in general stroke biology and weaker for direct causation of clinical PSS. These pathways should be framed as candidate phenotype modifiers rather than established determinants of PSS. Evidence categories distinguish direct human PSS evidence, direct post-stroke preclinical evidence, broader stroke evidence, and comparator-model evidence.

## 5 Mechanistic interfaces between mitochondrial–neuroimmune injury and post-stroke spasticity

### 5.1 From parallel mechanisms to interfaces

The purpose of this interface model is not to replace established neural mechanisms, but to identify where mitochondrial and neuroimmune processes may modify circuit and muscle remodeling after stroke. These candidate modifiers may influence the probability, severity, persistence, and clinical expression of spastic motor phenotypes.

### 5.2 Injured neurovascular unit and maladaptive motor-network plasticity

The first interface lies in the injured neurovascular unit and the broader motor-network recovery environment. Stroke-induced mitochondrial dysfunction and neuroimmune activation occur in neurons, astrocytes, microglia, endothelial cells, pericytes, oligodendrocyte-lineage cells, and infiltrating immune cells. These cellular responses influence tissue survival, synaptic stability, myelin integrity, axonal repair, angiogenesis, and the balance between inflammatory persistence and resolution. Neuroimmune and inflammatory factors can influence cellular, synaptic, structural, and anatomical plasticity after CNS injury, making them relevant to post-stroke circuit remodeling even when they are not specific to spasticity (O’Reilly and Tom, 2020; Qiao et al., 2023).

A useful way to frame this mechanism is to separate plasticity capacity from plasticity direction. Mitochondrial and immune responses may support repair when they promote debris clearance, metabolic stabilization, synaptic remodeling, and resolution of inflammation. The same broad response may become maladaptive when mitochondrial damage, lipid accumulation, oxidative stress, or persistent glial activation sustains inflammatory signaling and interferes with white matter repair or network refinement (Zhao et al., 2025).

### 5.3 Brainstem–spinal interface: descending drive, chloride homeostasis, and inhibitory remodeling

The second interface lies in the brainstem–spinal axis. Established models already implicate corticoreticulospinal imbalance, enhanced reticulospinal influence, altered spinal sensory processing, and motoneuron hyperexcitability in PSS. The mitochondrial–neuroimmune framework adds a cellular question: can inflammatory or metabolic signals after stroke modify spinal inhibitory control and thereby facilitate reflex amplification?

Within this framework, KCC2 represents a plausible molecular bridge between supraspinal injury and spinal disinhibition, but it is not an established pathway in human PSS. In a mouse model of post-stroke spasticity, Toda and colleagues reported decreased KCC2 expression and reduced S940 phosphorylation in the plasma membrane of spinal motoneurons during the early post-stroke period, together with increased vGluT1-positive boutons on spinal motoneurons at later time points (Toda et al., 2014). This study is valuable because it directly connects cerebral ischemic injury to spinal molecular remodeling. Stronger causal evidence for KCC2 and spasticity comes from spinal cord injury models, where KCC2 downregulation reduces postsynaptic inhibition and contributes to spasticity-like physiology (Boulenguez et al., 2010).

Microglia–BDNF–KCC2 signaling is another attractive comparator mechanism. In neuropathic pain models, ATP-stimulated microglia can signal to neurons through BDNF and collapse the neuronal anion gradient, thereby causing disinhibition (Coull et al., 2005). Enhancement of KCC2 activity has also been reported to decrease hyperreflexia and spasticity in chronic spinal cord injury models (Bilchak et al., 2021). These mechanisms are not PSS-specific, but they provide biologically plausible routes by which neuroimmune activation could alter inhibitory tone. Thus, the current evidence hierarchy consists of limited post-stroke animal evidence, supported by comparator evidence from spinal cord injury and pain models, without direct confirmation in human PSS.

### 5.4 Skeletal muscle interface: mitochondrial dysfunction, autophagy, stiffness, and contracture

The third interface is the peripheral skeletal muscle. Chronic post-stroke hypertonia is rarely a purely neural phenomenon. Reflex hyperexcitability, involuntary descending drive, spastic dystonia, pain, muscle shortening, extracellular matrix remodeling, tendon changes, and fixed contracture can all contribute to resistance during passive movement. A mitochondrial–neuroimmune framework is useful because skeletal muscle is a metabolically active tissue that responds to reduced activation, disuse, inflammation, altered loading, and systemic metabolic stress.

Post-stroke skeletal muscle remodeling is well documented. Human and review evidence describes muscle atrophy, altered fiber-type composition, reduced oxidative capacity, intramuscular fat accumulation, weakness, impaired endurance, and changes in inflammatory or metabolic markers (Azzollini et al., 2021; Beckwée et al., 2022; Hafer-Macko et al., 2008; Qi et al., 2023; Severinsen et al., 2016). In a rat MCAO model, ischemic stroke was associated with reductions in soleus tissue weight, pulling force, exercise capacity, endurance, and muscle structure, supporting a brain–muscle pathological consequence of stroke (Qi et al., 2023). These findings do not prove that muscle mitochondrial dysfunction causes spasticity, but they support the idea that stroke can induce peripheral tissue pathology capable of modifying chronic motor phenotypes.

Muscle mitochondrial dysfunction, fibrosis, and extracellular matrix remodeling do not constitute spasticity in the strict neurophysiological sense. They may increase passive resistance, reduce tissue compliance, and promote contracture, thereby amplifying the non-neural component of spastic hypertonia. Consequently, clinician-rated resistance may increase even when reflex excitability is not the dominant driver (Beckwée et al., 2022; Stecco et al., 2014).

### 5.5 Systemic inflammatory and metabolic modifiers

The fourth interface is systemic. Stroke occurs in patients with variable age, sex, vascular risk factors, diabetes, obesity, sarcopenia, infection burden, frailty, physical activity history, medication exposure, and rehabilitation access. These systemic variables influence mitochondrial function, immune tone, skeletal muscle composition, vascular health, and recovery capacity. They may not determine PSS alone, but they may modify the trajectory from acute motor pathway injury to chronic spastic phenotype.

This systemic interface can help explain clinical heterogeneity. Two patients with similar corticospinal injury may develop different spastic phenotypes if one has severe paresis, prolonged immobilization, diabetes, recurrent infection, low rehabilitation dose, sarcopenia, and high inflammatory burden, whereas the other receives early mobilization and maintains better metabolic reserve. The goal is not to force all phenotypes into one model, but to provide a structured way to study heterogeneity.

### 5.6 Integrative model and limits

These interfaces can be integrated into a brain–spinal cord–muscle axis. In the injured brain, mitochondrial stress and neuroimmune activation influence neuronal survival, glial state, vascular stability, synaptic remodeling, and white matter repair. In descending motor systems, corticospinal and corticoreticulospinal disruption alters the balance between voluntary control and brainstem-mediated facilitation. In the spinal cord, altered descending drive interacts with inhibitory dysfunction, chloride homeostasis, afferent input, and motoneuron excitability. In skeletal muscle, reduced activation, inflammation, mitochondrial dysfunction, autophagy-related remodeling, extracellular matrix changes, and tendon shortening increase non-neural resistance.

The model does not treat mitochondrial dysfunction as a primary cause of PSS or present mitochondrial–immune markers and therapies as clinically validated; rather, it defines testable candidate modifiers of circuit and muscle remodeling.

## 6 Mechanism-informed phenotyping: feasible assessments and exploratory biomarkers

### 6.1 Why phenotyping matters

A mechanism-oriented review of PSS requires a phenotyping strategy that can distinguish related but non-identical contributors to hypertonia. A patient with increased resistance during passive elbow extension may have stretch reflex hyperexcitability, spastic dystonia, enhanced reticulospinal drive, impaired reciprocal inhibition, pain-related guarding, muscle shortening, increased extracellular matrix stiffness, or fixed contracture. These mechanisms can coexist, but they have different biological origins and different treatment implications.

A useful framework should combine clinical examination, neurophysiology, structural and tract imaging, muscle mechanical assessment, and exploratory molecular biomarkers. Each domain captures a different level of the brain–spinal cord–muscle axis. No single domain is sufficient, but together they could support biologically stratified PSS research. For clarity, the following measures are considered in two tiers: currently feasible clinical or experimental phenotyping tools, and exploratory molecular markers that are not ready for clinical decision-making.

### 6.2 Clinical scales: useful for severity, limited for mechanism

Clinical scales remain necessary because they are practical and widely used. The Modified Ashworth Scale is the most common bedside tool for grading increased resistance to passive movement; the Modified Tardieu Scale adds a more explicitly velocity-dependent assessment by comparing muscle response at different stretch velocities and recording the angle of catch or clonus. However, common clinical scales have limitations in reliability, reproducibility, and ability to separate neural from non-neural components of resistance (Gal et al., 2025; He et al., 2023).

MAS and MTS should therefore be interpreted as clinical severity measures, not mechanism-resolving biomarkers. A high MAS score cannot determine whether resistance is dominated by reflex-mediated activation, spastic dystonia, passive muscle stiffness, tendon shortening, pain, or contracture. This limitation is particularly problematic in trials that aim to test mitochondrial, inflammatory, neuromodulatory, or muscle-targeted interventions.

### 6.3 EMG, h-reflex, TMS, and reticulospinal probes

Surface or intramuscular EMG during passive stretch can help distinguish active reflex-mediated muscle activation from passive mechanical resistance. This is essential because strict spasticity is velocity-dependent stretch reflex exaggeration, whereas chronic spastic hypertonia often includes substantial non-neural resistance (Hoffmann et al., 2009; McDonough et al., 2026). H-reflex testing provides a non-invasive probe of spinal reflex excitability, particularly in lower-limb muscles, but is highly context-sensitive and varies with posture, background activation, joint position, stimulation parameters, and chronicity after stroke (Frenkel-Toledo et al., 2021; Lamy et al., 2009; Qin et al., 2021).

Transcranial magnetic stimulation can assess corticospinal excitability, motor threshold, motor-evoked potential amplitude, silent period, and intracortical inhibition or facilitation. TMS contributes to mechanism-informed stratification, but it does not directly measure reticulospinal output, spinal reflex gain, muscle stiffness, or mitochondrial–immune status. StartReact paradigms provide one of the few practical human approaches for probing reticulospinal contribution after stroke, although they are indirect and should be interpreted alongside CST/CRT imaging, clinical phenotype, and EMG (Choudhury et al., 2019).

### 6.4 Imaging and muscle mechanics

Imaging is essential for linking phenotype to anatomy. Conventional MRI can identify lesion location, volume, stroke subtype, corticospinal involvement, and secondary degeneration. Diffusion MRI and tractography can provide information about corticospinal and corticoreticular integrity, (Ko et al., 2021) and emerging brainstem or spinal cord imaging may improve assessment of alternate motor pathways after stroke (Karbasforoushan et al., 2019; Oquita et al., 2024). Lesion and tract imaging can identify anatomical vulnerability to spasticity, but imaging alone cannot define the physiological state of spinal reflexes or the mechanical state of muscle.

Muscle ultrasound and shear-wave elastography are particularly important because they quantify the peripheral component of post-stroke hypertonia. Ultrasound can assess muscle thickness, architecture, echogenicity, and structural changes, whereas shear-wave elastography can quantify tissue stiffness. Ultrasound elastography has been described as promising for determining muscle stiffness in stroke survivors, and a recent meta-analysis reported a moderate association between shear-wave elastography-derived stiffness and clinician-rated spasticity scales (Kim et al., 2026; Roots et al., 2022).

### 6.5 Exploratory inflammatory and mitochondrial biomarkers

Candidate molecular biomarkers are attractive because they could, in principle, identify biological states that precede or modify PSS. Potential markers include circulating cytokines and chemokines, oxidative stress markers, antioxidant capacity, cell-free nuclear DNA, cell-free mitochondrial DNA, immune-cell metabolic signatures, mitophagy-related proteins, extracellular vesicle cargo, and muscle-derived molecular markers. However, none should currently be described as validated biomarkers for PSS.

The stroke biomarker literature supports cautious exploration. Inflammatory biomarkers and oxidative stress markers have been widely studied after ischemic stroke, but clinical use remains limited by timing, heterogeneity, and lack of specificity (Pawluk et al., 2024; Tirandi et al., 2023). Serial plasma nuclear and mitochondrial DNA levels have been reported to reflect cerebral damage severity after acute cerebral infarction, supporting their potential as injury-related markers (Tsai et al., 2011). For PSS, any molecular biomarker must be interpreted in a multimodal model that includes lesion and tract injury, neurophysiology, muscle stiffness, pain, and rehabilitation exposure.

### 6.6 Proposed longitudinal phenotyping design

**FIGURE 2:**
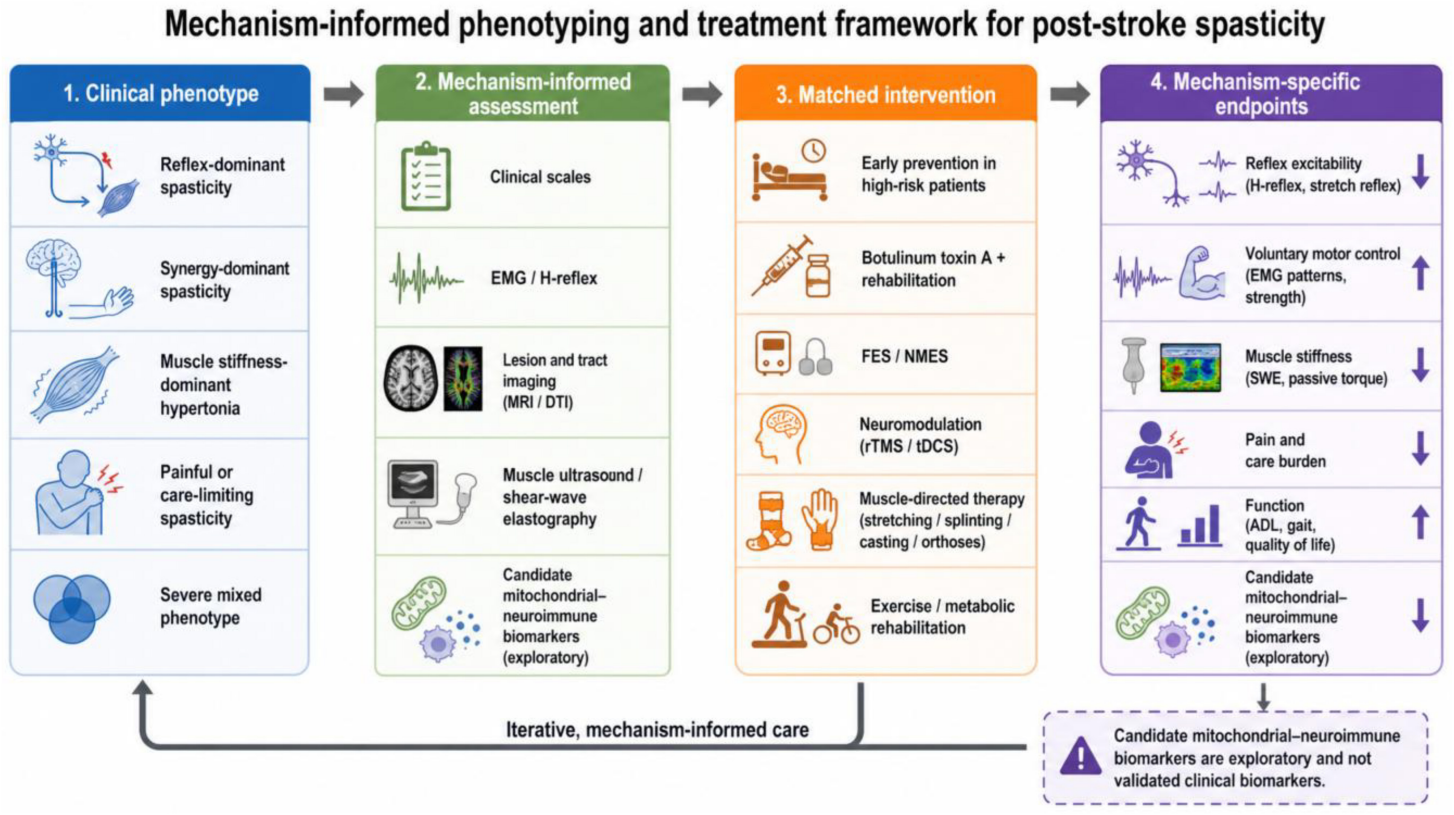
Proposed mechanism-informed phenotyping and treatment framework for post-stroke spasticity. A mechanism-informed approach begins by defining the dominant clinical phenotype rather than relying on tone severity alone. Clinically feasible or experimentally established assessments—including clinical scales, neurophysiology, lesion and tract imaging, and muscle mechanical assessment—should be distinguished from exploratory mitochondrial–neuroimmune biomarkers. Interventions should then be matched to the dominant mechanism and disease stage. Established or clinically grounded options include focal chemodenervation, rehabilitation, electrical stimulation, stretching, splinting, and selected neuromodulation approaches; mitochondrial- or immune-targeted strategies remain investigational hypotheses for future studies. Conceptual framework; not a validated clinical decision algorithm. Candidate mitochondrial–neuroimmune biomarkers remain exploratory and should not be interpreted as validated clinical biomarkers for PSS.

The strongest future design would be longitudinal rather than cross-sectional. Patients with acute or early subacute stroke and motor deficits should be enrolled early and followed across the period when spasticity emerges. Baseline and follow-up assessments should include lesion imaging, CST and CRT integrity, early paresis severity, sensory loss, inflammatory and mitochondrial candidate markers, infection status, diabetes and metabolic comorbidity, rehabilitation dose, and serial motor phenotype measures. At follow-up, the same cohort should undergo clinical scales, EMG during passive stretch, H-reflex testing, TMS or StartReact where feasible, ultrasound/SWE, range of motion, pain scales, function measures, and goal-based outcomes. Such designs would determine whether mitochondrial–neuroimmune markers add predictive value beyond established risk factors and whether they identify biologically distinct PSS subgroups. This phenotyping-to-intervention logic is summarized in Figure 2.

## 7 Translational implications: mechanism-based management rather than tone reduction alone

### 7.1 From antispastic treatment to mechanism-informed care

The clinical management of PSS has traditionally been organized around reducing muscle overactivity, improving range of motion, facilitating hygiene and positioning, relieving pain, and supporting function. These goals remain essential. However, a mechanism-informed framework suggests that treatment should not be reduced to suppression of tone alone. PSS can reflect different combinations of altered descending drive, spinal reflex hyperexcitability, spastic dystonia, passive muscle stiffness, pain, contracture, impaired voluntary control, and systemic inflammatory or metabolic modifiers.

Current evidence does not justify mitochondrial-targeted or immune-targeted therapy as an established treatment for PSS. Instead, the translational value of the framework lies in earlier risk recognition, matching interventions to dominant mechanisms, and designing trials that use endpoints beyond clinical tone scales. A recent American Heart Association scientific statement emphasized early recognition and intervention for poststroke spasticity and defined early intervention as treatment initiated within the first 3 months after stroke (Bandela et al., 2026). For clarity, Sections 7.2–7.4 summarize clinically grounded management approaches, whereas section “7.5 Investigational mitochondrial and neuroimmune targets” presents investigational hypotheses for future mechanistic studies and trials.

### 7.2 Early recognition and stage-specific intervention

The delayed evolution of PSS creates an opportunity for early recognition. Early paresis, sensory impairment, severe motor deficit, lesion location, corticospinal or corticoreticular tract injury, reduced mobility, and early increases in passive resistance can help identify patients who may be at risk for later disabling spasticity. Early intervention should be interpreted broadly: positioning, mobilization, range-of-motion maintenance, pain management, task-specific training, prevention of learned non-use, early identification of focal overactivity, and timely referral to a multidisciplinary spasticity service (Bandela et al., 2026; Francisco and McGuire, 2012).

### 7.3 Botulinum toxin A and focal spasticity management

Botulinum toxin A remains one of the most important established treatments for focal PSS. It reduces acetylcholine release at the neuromuscular junction and thereby decreases focal muscle overactivity. Intramuscular BoNT-A is widely used as a focal, reversible treatment for post-stroke muscle overactivity and is supported by clinical guidance and real-world practice data (Francisco and McGuire, 2012; Levy et al., 2023). For this Review, BoNT-A should be discussed not only as a tone-reducing therapy but also as an enabling intervention that may facilitate hygiene, positioning, pain control, brace tolerance, gait mechanics, hand opening, and rehabilitation participation.

Evidence increasingly supports combined approaches, including early BoNT-A or BoNT-A combined with rehabilitation, although study heterogeneity remains substantial (Ke et al., 2024; van Tilborg et al., 2025). BoNT-A reduces the peripheral expression of focal muscle overactivity and may enable more effective rehabilitation, but it should not be expected to normalize descending pathway imbalance, spinal reflex physiology, or chronic muscle remodeling when used in isolation.

### 7.4 Rehabilitation, electrical stimulation, and neuromodulation

Rehabilitation is the main clinical tool for shaping activity-dependent plasticity after stroke. Task-specific practice, strengthening, stretching, gait training, upper-limb training, balance training, and aerobic or combined exercise may influence voluntary motor control, activity-dependent plasticity, muscle oxidative capacity, systemic inflammatory tone, and contracture risk. These effects are highly relevant to chronic PSS, but they should be evaluated with endpoints that capture function, reflex excitability, muscle stiffness, pain, endurance, and participation rather than MAS alone.

Functional electrical stimulation and neuromuscular electrical stimulation occupy an important position between neural and peripheral treatment. Depending on protocol, stimulation can activate paretic muscles, provide sensory input, assist task practice, reduce antagonist overactivity, increase range of motion, and support gait or upper-limb function (Khan et al., 2023; Stein et al., 2015). Non-invasive brain stimulation, including rTMS and tDCS, has been investigated as a way to modulate cortical excitability and descending control; evidence is promising but heterogeneous, and patient selection remains unresolved (Fan et al., 2022; Wang et al., 2022).

### 7.5 Investigational mitochondrial and neuroimmune targets

Mitochondrial- and immune-targeted approaches are not current treatments for PSS. They are discussed here solely as hypotheses for mechanistically designed preclinical studies or biomarker-enriched clinical trials. Stroke research has generated interest in mitochondrial-targeted antioxidants, permeability-transition modulation, mitophagy regulators, mitochondrial transfer, anti-inflammatory strategies, inflammasome inhibition, STING pathway modulation, and immune-metabolic therapies; however, the evidence is derived mainly from general stroke injury, neuroprotection, repair biology, or preclinical models rather than clinically phenotyped PSS (Olaru et al., 2025).

Their most immediate role is trial design. A study might test whether exercise-based rehabilitation improves muscle oxidative capacity and reduces stiffness in chronic PSS, or whether early inflammatory or mitochondrial biomarkers identify patients at risk for severe spastic hypertonia. A preclinical study might test whether NLRP3 or STING modulation alters spinal inhibitory remodeling after stroke, but only if it measures stretch reflex physiology and spasticity-like outcomes rather than infarct volume alone.

### 7.6 Endpoint selection

Endpoint selection should follow mechanism. A reduction in MAS may show that passive resistance has changed, but it does not reveal whether the change reflects reduced reflex activation, reduced resting overactivity, altered passive stiffness, improved pain, or examiner variability. Trials that claim mechanism-based effects should include mechanism-based endpoints. This is particularly important for mitochondrial and neuroimmune interventions, where biological plausibility will remain weak unless molecular changes are linked to circuit, muscle, and functional outcomes. Table 3 summarizes assessment and intervention strategies aligned with this mechanism-informed approach.

**TABLE 3:** Mechanism-informed assessment and intervention strategy for post-stroke spasticity.

Dominant therapeutic target	Representative strategies	Mechanism-specific endpoints	Main translational implication
Early risk and secondary prevention	Early recognition, positioning, mobilization, range-of-motion preservation, pain control	Early paresis, lesion/tract injury, range of motion, emerging tone, pain, rehabilitation dose	Prevent progression from early neural overactivity to chronic mixed hypertonia
Focal muscle overactivity	Botulinum toxin A, focal chemodenervation combined with rehabilitation	Goal attainment, focal tone, EMG overactivity, pain, hygiene, function	Tone reduction should be used to enable training, care, and functional goals
Circuit-level maladaptive plasticity	Task-specific rehabilitation, FES/NMES, rTMS/tDCS in selected patients	Motor function, EMG, TMS, H-reflex, gait or upper-limb kinematics	Interventions should target voluntary control and reflex regulation, not tone alone
Non-neural muscle stiffness and contracture risk	Stretching, splinting, casting, orthoses, muscle-directed therapy	Range of motion, passive torque, ultrasound, shear-wave elastography, pain	Muscle mechanics should be measured separately from reflex-mediated spasticity
Systemic and metabolic vulnerability	Exercise, strengthening, aerobic training, physical conditioning	Endurance, strength, fatigue, activity level, muscle oxidative/metabolic markers	Muscle and systemic health may modify chronic spastic hypertonia and rehabilitation responsiveness
Investigational mitochondrial–neuroimmune targets (research settings only)	Antioxidant, mitophagy, inflammasome, STING, or immune-metabolic strategies in research settings	Candidate biomarkers plus neurophysiology, muscle stiffness, and function	These approaches are investigational and should not be presented as established PSS therapies

MAS and MTS remain useful clinical scales, but mechanism-oriented trials should pair them with endpoints that distinguish reflex excitability, descending control, muscle stiffness, pain, and function. BoNT-A, botulinum toxin A; EMG, electromyography; FES, functional electrical stimulation; MAS, Modified Ashworth Scale; MTS, Modified Tardieu Scale; NMES, neuromuscular electrical stimulation; PSS, post-stroke spasticity; rTMS, repetitive transcranial magnetic stimulation; tDCS, transcranial direct current stimulation; TMS, transcranial magnetic stimulation. The first five rows summarize clinically grounded or currently feasible strategies; the final row presents hypothesis-generating research directions rather than current PSS treatment.

## 8 Discussion and future directions

### 8.1 Summary of the proposed framework

PSS is best understood as a chronic motor phenotype that emerges from interactions among lesion anatomy, descending pathway imbalance, spinal reflex remodeling, and peripheral tissue adaptation. The most established mechanisms remain neural: damage to corticospinal and corticoreticulospinal projections, altered brainstem descending drive, increased spinal reflex gain, impaired inhibitory control, and motoneuron hyperexcitability. The central argument of this Review is not that mitochondrial dysfunction replaces these mechanisms. Rather, mitochondrial stress and neuroimmune activation may define the biological conditions in which motor circuits and skeletal muscle remodel after stroke.

### 8.2 What is established and what is plausible

Several conclusions can be stated with reasonable confidence. PSS is a clinically important but heterogeneous manifestation of the upper motor neuron syndrome. The strongest mechanistic foundation remains altered neural control. Stroke induces robust mitochondrial and neuroimmune responses. Skeletal muscle after stroke undergoes structural, metabolic, and mechanical remodeling that may increase non-neural resistance. These areas provide the foundation for a brain–spinal cord–muscle axis.

The more speculative part concerns how mitochondrial–neuroimmune mechanisms interface with PSS-specific mechanisms. Links between mitochondrial–immune signaling and maladaptive motor-network plasticity, spinal inhibitory remodeling, mitochondrial danger signaling, and peripheral muscle remodeling are biologically plausible but incompletely tested. These distinctions should be preserved throughout the final manuscript: established PSS mechanisms, strong stroke mitochondrial–immune biology, and hypothesis-generating interfaces should not be conflated.

### 8.3 Priority research directions

The most urgent research need is longitudinal human phenotyping. Acute or early subacute stroke cohorts should be followed through the window in which PSS emerges and consolidates. Essential data include lesion location and volume, CST/CRT integrity, paresis severity, sensory impairment, pain, infection, diabetes, systemic inflammatory status, medication exposure, rehabilitation dose, clinical spasticity scales, stretch-evoked EMG, H-reflex or other spinal excitability measures, TMS or StartReact where feasible, ultrasound/SWE, range of motion, pain, motor function, participation, and candidate mitochondrial or inflammatory biomarkers.

A second priority is preclinical modeling that measures spasticity-like outcomes, not infarct volume alone. Future animal studies should include longitudinal stretch reflexes, EMG responses to passive movement, motoneuron excitability, H-reflex modulation, spinal inhibitory markers, afferent synaptic remodeling, muscle stiffness, and muscle histology. Mitochondrial and immune perturbations should be linked to these endpoints rather than to lesion volume alone.

A third priority is cell-type-specific and compartment-specific profiling. Mitochondrial injury in neurons, microglia, astrocytes, endothelial cells, infiltrating leukocytes, spinal motoneurons, and skeletal muscle has different causes and consequences. Future studies should adopt single-cell, spatial, electrophysiological, imaging, and muscle-level approaches to define which cell type and anatomical compartment are relevant to each phenotype component.

A fourth priority is better assessment of alternate motor pathways and spinal cord mechanisms. CST integrity is essential for motor outcome, but PSS likely involves corticoreticulospinal, reticulospinal, vestibulospinal, rubrospinal, propriospinal, and spinal mechanisms in varying combinations. Imaging and functional assays should be combined to define patient-level mechanism profiles.

### 8.4 Concluding perspective

The most defensible conclusion is that mitochondrial stress and neuroimmune activation are best regarded at present as candidate modifiers of phenotype trajectory rather than simple causes of spasticity. They may influence whether post-stroke plasticity supports recovery or consolidates maladaptive motor patterns, whether spinal inhibitory circuits become persistently hyperexcitable, whether skeletal muscle develops stiffness and metabolic dysfunction, and whether systemic inflammatory or metabolic states increase vulnerability to disabling hypertonia. By integrating mitochondrial–immune biology with established circuit and muscle mechanisms, the field may move toward earlier risk stratification, more precise phenotyping, and intervention strategies that target the dominant mechanisms of disability rather than tone alone.

## References

[B1] Azzollini V. Dalise S. Chisari C. (2021). How does stroke affect skeletal muscle? State of the art and rehabilitation perspective. *Front. Neurol.* 12:797559. 10.3389/fneur.2021.797559 35002937 PMC8733480

[B2] Bandela S. McPherson L. Harvey R. Awosika O. Aggarwal D. Liu C. et al.. (2026). Early recognition and intervention for poststroke spasticity: A scientific statement from the American Heart Association. *Stroke* 57 e146–e159. 10.1161/STR.0000000000000515 41608795 PMC13063836

[B3] Beckwée D. Lefeber N. Bautmans I. Cuypers L. De Keersmaecker E. Kerckhofs E. et al.. (2022). Skeletal muscle changes in the first three months of stroke recovery: A systematic review. *J. Rehabil. Med.* 54:jrm00267. 10.2340/jrm.v54.573 35848335 PMC9575591

[B4] Bellut M. Papp L. Bieber M. Kraft P. Stoll G. Schuhmann M. (2023). Delayed NLRP3 inflammasome inhibition ameliorates subacute stroke progression in mice. *J. Neuroinflammation* 20:23. 10.1186/s12974-022-02674-w 36600259 PMC9811791

[B5] Bilchak J. Caron G. Côté M. (2021). Enhancing KCC2 activity decreases hyperreflexia and spasticity after chronic spinal cord injury. *Exp. Neurol.* 338:113605. 10.1016/j.expneurol.2021.113605 33453210 PMC7904648

[B6] Billingham L. Stoolman J. Vasan K. Rodriguez A. Poor T. Szibor M. et al.. (2022). Mitochondrial electron transport chain is necessary for NLRP3 inflammasome activation. *Nat. Immunol.* 23 692–704. 10.1038/s41590-022-01185-3 35484407 PMC9098388

[B7] Boulenguez P. Liabeuf S. Bos R. Bras H. Jean-Xavier C. Brocard C. et al.. (2010). Down-regulation of the potassium-chloride cotransporter KCC2 contributes to spasticity after spinal cord injury. *Nat. Med.* 16 302–307. 10.1038/nm.2107 20190766

[B8] Burke D. Wissel J. Donnan G. (2013). Pathophysiology of spasticity in stroke. *Neurology* 80 S20–S26. 10.1212/WNL.0b013e31827624a7 23319482

[B9] Choudhury S. Shobhana A. Singh R. Sen D. Anand S. Shubham S. et al.. (2019). The relationship between enhanced reticulospinal outflow and upper limb function in chronic stroke survivors. *Neurorehabil Neural Repair.* 33 375–383. 10.1177/1545968319836233 30913964

[B10] Coull J. Beggs S. Boudreau D. Boivin D. Tsuda M. Inoue K. et al.. (2005). BDNF from microglia causes the shift in neuronal anion gradient underlying neuropathic pain. *Nature* 438 1017–1021. 10.1038/nature04223 16355225

[B11] Dietz V. Sinkjær T. (2007). Spastic movement disorder: Impaired reflex function and altered muscle mechanics. *Lancet Neurol.* 6 725–733. 10.1016/S1474-4422(07)70193-X 17638613

[B12] Fan J. Fu H. Xie X. Zhong D. Li Y. Liu X. et al.. (2022). The effectiveness and safety of repetitive transcranial magnetic stimulation on spasticity after upper motor neuron injury: A systematic review and meta-analysis. *Front. Neural Circuits* 16:973561. 10.3389/fncir.2022.973561 36426136 PMC9679509

[B13] Francisco G. McGuire J. (2012). Poststroke spasticity management. *Stroke* 43 3132–3136. 10.1161/STROKEAHA.111.639831 22984012

[B14] Frenkel-Toledo S. Bentin S. Perry A. Liebermann D. Soroker N. (2021). Dynamics of the stretch reflex threshold in post-stroke spasticity. *J. Neuroeng. Rehabil.* 18:28. 33985543 10.1186/s12984-021-00876-6 PMC8117272

[B15] Gal O. Baude M. Deltombe T. Esquenazi A. Gracies J. Hoskovcova M. et al.. (2025). Clinical outcome assessments for spasticity: Review, critique, and recommendations. *Mov. Disord.* 40 22–43. 10.1002/mds.30062 39629752 PMC11752990

[B16] Gao L. Zheng M. Wang T. Zhang J. Li H. Wang Z. (2024). Mitochondrial stress: A key role of neuroinflammation in stroke. *J. Neuroinflammation* 21:44. 10.1186/s12974-024-03033-7 38321473 PMC10845693

[B17] Hafer-Macko C. Ryan A. Ivey F. Macko R. (2008). Skeletal muscle changes after hemiparetic stroke and potential beneficial effects of exercise intervention strategies. *J. Rehabil. Res. Dev.* 45 261–272. 10.1682/JRRD.2007.02.0040 18566944 PMC2978978

[B18] He J. Zhang Y. Wang W. Ding Y. (2023). Quantitative assessment of spasticity after stroke: A narrative review. *Front. Neurol.* 14:1123914. 10.3389/fneur.2023.1121323 PMC10354649 37475737

[B19] Hoffmann G. Kamper D. Kahn J. Rymer W. Schmit B. (2009). Modulation of stretch reflexes of the finger flexors by sensory feedback from the proximal upper limb poststroke. *J. Neurophysiol.* 102 1420–1429. 10.1152/jn.90950.2008 19571191 PMC2746792

[B20] Iadecola C. Anrather J. (2011). The immunology of stroke: From mechanisms to translation. *Nat. Med.* 17 796–808. 10.1038/nm.2399 21738161 PMC3137275

[B21] Iadecola C. Buckwalter M. Anrather J. (2020). Immune responses to stroke: Mechanisms, modulation, and therapeutic potential. *J. Clin. Invest.* 130 2777–2788. 10.1172/JCI135530 32391806 PMC7260029

[B22] Karbasforoushan H. Cohen-Adad J. Dewald J. (2019). Brainstem and spinal cord MRI identifies altered sensorimotor pathways post-stroke. *Nat. Commun.* 10:3524. 10.1038/s41467-019-11244-3 31388003 PMC6684621

[B23] Katz R. Rymer W. (1989). Spastic hypertonia: Mechanisms and measurement. *Arch. Phys. Med. Rehabil.* 70 144–155. 2644919

[B24] Ke M. Li D. Zhou P. Geng H. Zhang Q. Zhi L. et al.. (2024). Efficacy of botulinum toxin combined with rehabilitation treatments in the treatment of post-stroke spasticity: A systematic review and network meta-analysis. *NeuroRehabilitation* 55 399–416. 10.1177/10538135241290110 42543864

[B25] Khan M. Fares H. Ghaffar A. Meador K. Song R. Lin C. (2023). A systematic review on functional electrical stimulation-based rehabilitation systems for upper limb post-stroke therapy. *Front. Neurol.* 14:1272992. 10.3389/fneur.2023.1272992 38145118 PMC10739305

[B26] Kim J. Oh S. Kim S. Kim T. Kim Y. (2026). Clinical validity of shear wave elastography for post-stroke spasticity: A systematic review and meta-analysis. *J. Clin. Med.* 15:2063. 10.3390/jcm15052063 41827479 PMC12985620

[B27] Ko S. Park J. Lee P. Lee J. Park M. Bae J. (2021). Corticoreticular pathway in post-stroke spasticity: A diffusion tensor imaging study. *J. Pers. Med.* 11:1151. 10.3390/jpm11111151 34834503 PMC8621009

[B28] Kong L. Li W. Chang E. Wang W. Shen N. Wang H. et al.. (2022). mtDNA-STING axis mediates microglial polarization via IRF3/NF-κB signaling after ischemic stroke. *Front. Immunol.* 13:860977. 10.3389/fimmu.2022.860977 35450066 PMC9017276

[B29] Lamy J. Wargon I. Baret M. Ben Smail D. Milani P. Raoul S. et al.. (2005). Post-activation depression in various group I spinal pathways in humans. *Exp. Brain Res.* 166 248–262. 10.1007/s00221-005-2360-4 16078020

[B30] Lamy J. Wargon I. Mazevet D. Ghanim Z. Pradat-Diehl P. Katz R. (2009). Impaired efficacy of spinal presynaptic mechanisms in spastic stroke patients. *Brain* 132 734–748. 10.1093/brain/awn310 19036767

[B31] Lance J. (1980). “Symposium synopsis,” in *Spasticity: Disordered Motor Control*, eds Feldman R. Young R. Koella W. (Chicago: Year Book Medical Publishers), 485–494.

[B32] Lee-Hotta S. (2026). Involvement of the reticulospinal tract in the pathogenesis of spasticity after stroke and spinal cord injury. *Front. Neurosci.* 20:1753609. 10.3389/fnins.2026.1753609 41972249 PMC13066259

[B33] Levy J. Molteni F. Cannaviello G. Lansaman T. Roche N. (2023). Botulinum toxin use in patients with post-stroke spasticity: A nationwide retrospective study from France. *Front. Neurol.* 14:1245228. 10.3389/fneur.2023.1245228 37681005 PMC10482253

[B34] Li S. (2017). Spasticity, motor recovery, and neural plasticity after stroke. *Front. Neurol.* 8:120. 10.3389/fneur.2017.00120 28421032 PMC5377239

[B35] Li S. Francisco G. (2015). New insights into the pathophysiology of post-stroke spasticity. *Front. Hum. Neurosci.* 9:192. 10.3389/fnhum.2015.00192 25914638 PMC4392691

[B36] Liu W. Chen L. Zhang X. (2018). Mitochondria in ischemic stroke: New insight and implications. *Aging Dis.* 9 924–937. 10.14336/AD.2017.1126 30271667 PMC6147588

[B37] Lundström E. Terént A. Borg J. (2008). Prevalence of disabling spasticity 1 year after first-ever stroke. *Eur. J. Neurol.* 15 533–539. 10.1111/j.1468-1331.2008.02114.x 18355307

[B38] McArthur K. Whitehead L. Heddleston J. Li L. Padman B. Oorschot V. et al.. (2018). BAK/BAX macropores facilitate mitochondrial herniation and mtDNA efflux during apoptosis. *Science* 359:eaao6047. 10.1126/science.aao6047 29472455

[B39] McDonough M. Ludvig D. Ahmed Y. Perreault E. (2026). Reflex hyperexcitability persists during voluntary muscle activation following stroke. *J. Neurophysiol.* 135 1062–1071. 10.1152/jn.00543.2025 41893885

[B40] Olaru G. Buga A. Sandu R. Padureanu V. Popa D. Calina D. (2025). Harnessing mitochondrial function for post-stroke rehabilitation: Unlocking antioxidant power. *Antioxidants* 14:1080. 10.3390/antiox14091080 41008986 PMC12466533

[B41] Oquita R. Levy R. Newton J. Schambra H. (2024). Moving toward elucidating alternative motor pathway structure with the value of spinal cord neuroimaging in stroke-related motor dysfunction and recovery. *Front. Neurol.* 15:1282685. 10.3389/fneur.2024.1282685 38419695 PMC10899520

[B42] O’Reilly M. Tom V. (2020). Neuroimmune system as a driving force for plasticity following CNS injury. *Front. Cell. Neurosci.* 14:187. 10.3389/fncel.2020.00187 32792908 PMC7390932

[B43] Pawluk H. Wróbel M. Wróbel A. Jankowski M. (2024). The influence of oxidative stress markers in patients with stroke. *Antioxidants* 13:985. 10.3390/antiox13080985 39199231 PMC11351922

[B44] Qi H. Zhou J. Zhang Y. Li J. Wang L. Huang Y. et al.. (2023). Ischemic stroke induces skeletal muscle damage and alters transcriptome profile in rats. *J. Clin. Med.* 12:547. 10.3390/jcm12020547 36675476 PMC9865444

[B45] Qiao C. Xu Y. Chen Q. Lian J. Yang M. Dong Y. et al.. (2023). The implications of microglial regulation in neuroplasticity after ischemic stroke. *Biomolecules* 13:571. 10.3390/biom13030571 36979506 PMC10046452

[B46] Qin C. Yang S. Chu Y. Zhang H. Pang X. Chen L. et al.. (2022). Signaling pathways involved in ischemic stroke: Molecular mechanisms and therapeutic interventions. *Signal. Transduct Target. Ther.* 7:215. 10.1038/s41392-022-01064-1 35794095 PMC9259607

[B47] Qin W. Xiang X. Li X. Fan Y. Wang L. Zhang Z. (2021). Influence of body position on the H-reflex in post-stroke spasticity. *Front. Neurol.* 12:732963.

[B48] Rongvaux A. Jackson R. Harman C. Li T. West A. de Zoete M. et al.. (2014). Apoptotic caspases prevent the induction of type I interferons by mitochondrial DNA. *Cell* 159 1563–1577. 10.1016/j.cell.2014.11.037 25525875 PMC4272443

[B49] Roots J. Chen Y. Ball S. Chopp-Hurley J. Thain E. Hu B. et al.. (2022). Ultrasound elastography in the assessment of post-stroke muscle stiffness: A systematic review. *Insights Imaging* 13:67. 10.1186/s13244-022-01191-x 35380302 PMC8982789

[B50] Severinsen K. Jakobsen J. Overgaard K. Andersen H. (2016). Skeletal muscle fiber characteristics and oxidative capacity in hemiparetic stroke survivors. *Muscle Nerve* 53 748–754. 10.1002/mus.24907 26361074

[B51] Sheean G. McGuire J. (2009). Spastic hypertonia and movement disorders: Pathophysiology, clinical presentation, and quantification. *PM R.* 1 827–833. 10.1016/j.pmrj.2009.08.002 19769916

[B52] Shi K. Tian D. Li Z. Ducruet A. Lawton M. Shi F. (2019). Global brain inflammation in stroke. *Lancet Neurol.* 18 1058–1066. 10.1016/S1474-4422(19)30078-X 31296369

[B53] Shimada K. Crother T. Karlin J. Dagvadorj J. Chiba N. Chen S. et al.. (2012). Oxidized mitochondrial DNA activates the NLRP3 inflammasome during apoptosis. *Immunity* 36 401–414. 10.1016/j.immuni.2012.01.009 22342844 PMC3312986

[B54] Sommerfeld D. Eek E. Svensson A. Holmqvist L. von Arbin M. (2004). Spasticity after stroke: Its occurrence and association with motor impairments and activity limitations. *Stroke* 35 134–139. 10.1161/01.STR.0000105386.05173.5E 14684785

[B55] Stecco A. Stecco C. Raghavan P. (2014). Peripheral mechanisms contributing to spasticity and implications for treatment. *Curr. Phys. Med. Rehabil. Rep.* 2 121–127. 10.1007/s40141-014-0052-3

[B56] Stein C. Fritsch C. Robinson C. Sbruzzi G. Plentz R. (2015). Effects of electrical stimulation in spastic muscles after stroke: Systematic review and meta-analysis of randomized controlled trials. *Stroke* 46 2197–2205. 10.1161/STROKEAHA.115.009633 26173724

[B57] Tirandi A. Di Napoli M. Lubrano V. Ristori G. Orlandi G. (2023). Inflammatory biomarkers of ischemic stroke. *Intern. Emerg. Med.* 18 723–743. 10.1007/s11739-022-03139-x 36745280 PMC10082112

[B58] Toda T. Ishida K. Kiyama H. Yamashita T. Lee S. (2014). Down-regulation of KCC2 expression and phosphorylation in motoneurons, and increases the number of primary afferent projections to motoneurons in mice with post-stroke spasticity. *PLoS One* 9:e114328. 10.1371/journal.pone.0114328 25546454 PMC4278744

[B59] Trompetto C. Marinelli L. Mori L. Pelosin E. Curra A. Molfetta L. et al.. (2019). Spastic dystonia in stroke subjects: Prevalence and features of the neglected phenomenon of the upper motor neuron syndrome. *Clin. Neurophysiol.* 130 521–527. 10.1016/j.clinph.2019.01.012 30776732

[B60] Trompetto C. Marinelli L. Mori L. Pelosin E. Currà A. Molfetta L. et al.. (2014). Pathophysiology of spasticity: Implications for neurorehabilitation. *Biomed. Res. Int.* 2014:354906. 10.1155/2014/354906 25530960 PMC4229996

[B61] Tsai N. Lin T. Chen S. Chang W. Wang H. Yang T. et al.. (2011). The value of serial plasma nuclear and mitochondrial DNA levels in patients with acute ischemic stroke. *Clin. Chim. Acta* 412 476–479. 10.1016/j.cca.2010.11.029 21130757

[B62] van Tilborg N. de Groot V. Meskers C. (2025). The effectiveness of early interventions for post-stroke spasticity: A systematic review. *Disabil. Rehabil.* 47 900–911. 10.1080/09638288.2024.2363963 38907596

[B63] Wang X. Zhang Z. Hu H. Zhao J. Zheng X. (2022). Effects of non-invasive brain stimulation on post-stroke spasticity: A systematic review and meta-analysis. *Brain Sci.* 12:836. 10.3390/brainsci12070836 35884643 PMC9312973

[B64] West A. Khoury-Hanold W. Staron M. Tal M. Pineda C. Lang S. et al.. (2015). Mitochondrial DNA stress primes the antiviral innate immune response. *Nature* 520 553–557. 10.1038/nature14156 25642965 PMC4409480

[B65] White M. McArthur K. Metcalf D. Lane R. Cambier J. Herold M. et al.. (2014). Apoptotic caspases suppress mtDNA-induced STING-mediated type I IFN production. *Cell* 159 1549–1562. 10.1016/j.cell.2014.11.036 25525874 PMC4520319

[B66] Wissel J. Schelosky L. Scott J. Christe W. Faiss J. Mueller J. (2010). Early development of spasticity following stroke: A prospective, observational trial. *J. Neurol.* 257 1067–1072. 10.1007/s00415-010-5463-1 20140444 PMC2892615

[B67] Yang J. Mukda S. Chen S. (2022). Mitochondrial quality control: A pathophysiological mechanism and therapeutic target for stroke. *Front. Mol. Neurosci.* 14:786099. 10.3389/fnmol.2021.786099 35153669 PMC8832032

[B68] Zeng H. Chen J. Guo Y. Tan S. (2021). Prevalence and risk factors for spasticity after stroke: A systematic review and meta-analysis. *Front. Neurol.* 11:616097. 10.3389/fneur.2020.616097 33551975 PMC7855612

[B69] Zhao Q. Wang Y. Chen M. Shi Y. Liu S. Zhang J. et al.. (2025). Cholesterol metabolic reprogramming mediates microglia-induced chronic neuroinflammation and hinders neurorestoration following stroke. *Nat. Metab.* 7 2099–2116. 10.1038/s42255-025-01379-7 40987840 PMC12552130

[B70] Zhong Z. Liang S. Sanchez-Lopez E. He F. Shalapour S. Lin X. et al.. (2018). New mitochondrial DNA synthesis enables NLRP3 inflammasome activation. *Nature* 560 198–203. 10.1038/s41586-018-0372-z 30046112 PMC6329306

[B71] Zhou R. Yazdi A. Menu P. Tschopp J. (2011). A role for mitochondria in NLRP3 inflammasome activation. *Nature* 469 221–225. 10.1038/nature09663 21124315

